# Cannabinoid HU210 Protects Isolated Rat Stomach against Impairment Caused by Serum of Rats with Experimental Acute Pancreatitis

**DOI:** 10.1371/journal.pone.0052921

**Published:** 2012-12-28

**Authors:** Ming-hua Cao, Yong-yu Li, Jing Xu, Ya-jing Feng, Xu-hong Lin, Kun Li, Tong Han, Chang-Jie Chen

**Affiliations:** Institute of Digestive Diseases, Department of Pathophysiology, Tongji University School of Medicine, Shanghai, China; Nathan Kline Institute for Psychiatric Research and New York School of Medicine, United States of America

## Abstract

Acute pancreatitis (AP), especially severe acute pancreatitis often causes extra-pancreatic complications, such as acute gastrointestinal mucosal lesion (AGML) which is accompanied by a considerably high mortality, yet the pathogenesis of AP-induced AGML is still not fully understood. In this report, we investigated the alterations of serum components and gastric endocrine and exocrine functions in rats with experimental acute pancreatitis, and studied the possible contributions of these alterations in the pathogenesis of AGML. In addition, we explored the intervention effects of cannabinoid receptor agonist HU210 and antagonist AM251 on isolated and serum-perfused rat stomach. Our results showed that the AGML occurred after 5 h of AP replication, and the body homeostasis was disturbed in AP rat, with increased levels of pancreatic enzymes, lipopolysaccharide (LPS), proinflammtory cytokines and chemokines in the blood, and an imbalance of the gastric secretion function. Perfusing the isolated rat stomach with the AP rat serum caused morphological changes in the stomach, accompanied with a significant increment of pepsin and [H^+^] release, and increased gastrin and decreased somatostatin secretion. HU210 reversed the AP-serum-induced rat pathological alterations, including the reversal of transformation of the gastric morphology to certain degree. The results from this study prove that the inflammatory responses and the imbalance of the gastric secretion during the development of AP are responsible for the pathogenesis of AGML, and suggest the therapeutic potential of HU210 for AGML associated with acute pancreatitis.

## Introduction

Acute pancreatitis (AP), especially severe AP, is a potentially lethal inflammatory disease of pancreas which often leads to extra-pancreatic complications, even multiple systemic organ dysfunctions. It has been reported that 52% of patients with acute pancreatitis develop acute gastrointestinal mucosal lesion (AGML) or stress ulcer [1], [2]. Although the endoscopic observation shows that the majority of subjects merely have multiple shallow erosions in the gastrointestinal tract, the optimal pharmacological intervention continues to be a matter of debate, and the pathogenesis of AGML remains unclear.

Some investigators report that the stressful condition with acute pancreatitis causes the diminished blood supply or hypoperfusion in the gastric mucosa, and the counter-diffusion of gastric hydrogen ion (H^+^) is an important factor for AGML as well [3], [4]. Other investigations discovered that the serum and ascitic fluid from AP patients and experimental animals contained a large amount of toxic substances, such as pancreatic enzymes, endotoxins, inflammatory mediators [5], [6], which may contribute to the multiple organ dysfunctions in acute pancreatitis [7], [8].

For centuries, Cannabis plant and its extracts have been used to alleviate symptoms of gastrointestinal inflammatory diseases. It has been established that D^9^-tetrahydrocannabinol, the major psychoactive component of Cannabis, exerts its primary cellular actions though two G protein-coupled receptors, cannabinoid 1 (CB1) and cannabinoid 2 (CB2) receptors [9]–[11]. Since then, these two receptors have been recognized as the major regulators of physiological and pathological processes [12]. Cannabinoids can reduce gastrointestinal secretion [13], and the activation of CB1 receptor exhibits protective role against stress-induced AGML [14], [15], but the mechanisms of their action remain elusive.

The aim of the present work was to explore, by both in vivo and in vitro experiments, the changes in the serum components, the alterations of gastric endocrine and exocrine functions in rat AP model, and the possible contributions of these alterations in the pathogenesis of AGML. Also probed were the interventional effects of CB1 by using its agonist HU210 and antagonist AM251, in an effort to better elucidate the pathophysiological mechanisms of AP-associated AGML and the antiulcer potentials of these cannabinoid agents.

## Materials and Methods

### Animals

Male Sprague–Dawley rats (220–250 g) were obtained from the Experimental Animal Center of Fudan University, Shanghai, China. Prior to the experiments, all animals were housed for 1 week under standard conditions with free access to water and laboratory chow. All experimental procedures below were in agreement with international guidelines for the care and use of laboratory animals and were approved by the Animal Ethics Committee of Tongji University, Shanghai, China.

### Induction of Acute Pancreatitis in Rats

The rats were allocated randomly into two groups: AP and sham-operation group with 24 animals in each group. The rats were fasted overnight with only water allowed before surgery. AP model was induced by the method developed by Aho et al [16]. Briefly, the rats got laparotomy (∼3 cm abdominal-midline incision) following the standard aseptic procedure and under general anesthesia with intraperitoneal injection of 20% ethyl carbamate at 10 mL/kg. The biliopancreatic duct was temporarily occluded at the liver hilum with a fine soft microvascular clamp to prevent reflux of the infused material to the liver. A retrograde injection of 3% sodium deoxycholate into the biliopancreatic duct was then performed (0.1 mL/100 g bodyweight). The clamp was removed after the injection. Sham-operation was performed accordingly without the sodium deoxycholate injection, and the surgery was concluded with abdominal stratified closing. On the fifth hour after the surgery, the blood was collected from the abdominal aorta puncture under anaesthetization. All the samples of blood were centrifuged and the supernatant fluid (serum) was collected, aliquoted, and stored at −20°C for subsequent applications. The pancreas was removed, divided into two parts, and one part was put into trizol immediately and store at −20°C for genechip analysis, as the other part was fixed with 10% paraformaldehyde. The stomach was also removed, opened along the large curve, and fixed with 10% paraformaldehyde for ensuing pathological examination.

### Histological Evaluation

Histological evaluation was performed on rat pancreas and stomach that were fixed in 10% paraformaldehyde and embedded in paraffin. Thereafter, 5 µm thickness sections were sliced on a Leica RM2126 microtome (Leica, Shanghai, China) and stained with haematoxylin (0.5%) and eosin (0.5%), followed by observation under a Motic BA300 microscope (Motic China Group Co. Ltd., Xiamen, China). Histological Scoring was appraised on pancreatic sections using a modified criterion from Nathan JD, et al [17]. The evaluation was made in ten randomly chosen microscopic fields of each animal’s slides, and repeated in three rats /group in a blinded manner. And the total histological score (0–9) was expressed as the sum of edema (0–3), inflammatory cell infiltration (0–3), and tissue necrosis (0–3).

### Microarray Hybridization Assay

Microarray analysis was used to identify transcription profiles of some inflammatory indexes in the pancreas from rat with acute pancreatitis. Array hybridizations were carried out using three biological replicates of RNA samples extracted from the pancreas of AP and control rats. Probe preparation, chip hybridization, and primary data analysis were performed by Capital Bio Corporation (a firm licensed and authorized by Affymetrix to operate in Beijing, China). Arrays were scanned using the Genechip Scanner 3000 7G (Affymetrix, Santa Clara, CA, USA). Quantitative analysis was performed using Affymetric MicroArray Suite 5.0-Specific Terms (Statistical Algorithms) GCOS (Affymetrix GeneChip Operating Software) Version 1.4. The differentially expressed genes were identified using SAM (Significant Analysis of Microarray) software, and selected on the basis of their fold changes (>2-fold) as compared to the control specimens.

### Immunohistochemistry Analysis

Immunohistochemistry staining on paraffin sections of rat stomach and pancreas were performed using rabbit polyclonal anti-CB1 and anti-CB2 antibodies (Cat. no: ALX-210-314 for anti-CB1 and Cat. no: ALX-210-315 for anti-CB2, Enzo, Plymouth Meeting, PA, USA) as described previously [18]. The slides with sections of rat stomach and pancreas were incubated overnight at 4°C with anti-CB1 or anti-CB2 antibodies, and the biotin-labeled goat anti-rabbit IgG working fluid (Cat. no: SP0023; Biosynthesis Biotechnology Co. Ltd., Beijing, China) was then applied onto each slide and incubated at 37°C for 15 minutes, followed by incubation with a HRP-labeled streptavidin working solution at 37°C for 15 minutes, and slides were rinsed thoroughly. Finally, the slides were DAB-stained and nuclear re-stained with hematoxylin. The slides of the negative control were processed through the identical steps, but the primary antibody was replaced with PBS. Image analysis was accomplished using digital Motic Med 6.0 image analysis system (Motic; China Group Co. Ltd., Xiamen, China).

### Western Blotting for Measuring CB1 and CB2 Expression

CB1 and CB2 protein expression in the pancreas and stomach were evaluated by western blotting. As described previously [19], after incubation with the primary antibodies in a 1∶250 dilution individually (rabbit polyclonal anti-CB1 and anti-CB2 antibodies, Cat. no: ALX-210-314 for anti-CB1 and Cat. no: ALX-210-315 for anti-CB2, Enzo, Plymouth Meeting, PA, USA), the blotted nitrocellulose membranes (Whatman, Dassel, Germany) were rinsed thoroughly, and the appropriate secondary antibody conjugated to horseradish peroxidase was incubated for 1 hr at room temperature. For internal reference, polyclonal rabbit anti-mouse β-actin antibody (1∶2,000 dilution) (Abmart, Shanghai, China) was used. Finally, antibody binding was detected by exposure to ECL western blotting detection reagents (Cat. no: SC-2048, Santa Cruz Biotechnology, Santa Cruz, CA, USA) and recorded on film.

### Preparation of Isolated- vascularly Perfused Rat Stomach

Rat was anesthetized and the isolated, vascularly perfused rat stomach was prepared as described previously [20]. Briefly, the abdomen was opened with a midline incision under sterile condition. After ligation of the abdominal aorta just above the branching of the celiac artery, a cannula was inserted into the celiac artery via an incision placed on the aorta. Two milliliters of saline solution containing 600 U of heparin were then injected into the gastric artery via the arterial cannula. Subsequently, a warm (37°C) modified Krebs-Ringer solution bubbled with a mixture of 95% O_2_ and 5% CO_2_ was introduced. The venous effluent was collected via a portal vein cannula. A polyethylene tube for gastric lumen perfusate was inserted into the esophagus and the tip positioned in the luminal portion of the stomach. Afterward, the pyloroduodenal junction was exposed, and another polyethylene tube was introduced into the stomach via an incision on the duodenum, and then fixed by a ligature around the pylorus. The perfused rat stomach was isolated and placed in a warm (37°C) small chamber with Krebs-Ringer solution.

### Treatment of the Isolated Rat Stomach

The isolated stomach was vascularly perfused with modified Krebs-Ringer solution for 30 min equilibration before the formal experiments. The perfusion was then carried out sequentially with three fluids and each fluid for 20 minutes, totaling 60 minutes. The control group got: 1) Krebs-Ringer solution, 2) serum from normal control rats, 3) Krebs-Ringer solution. The AP group got: 1) Krebs-Ringer solution, 2) serum from AP rats, 3) Krebs-Ringer solution. The group of AP+HU got: 1) Krebs-Ringer solution+HU210 (10^−7^M), 2) AP serum+HU210 (10^−7^M), 3) Krebs-Ringer solution. And the group of AP+AM got: 1) Krebs-Ringer solution+AM251 (10^−7^M), 2) AP serum+AM251(10^−7^M), 3) Krebs-Ringer solution. The gastric lumen of the isolated stomach was perfused with normal saline (pH 7.0). All perfusion fluids ran at a constant rate of 1 ml/min by using micro-infusion pumps. Meanwhile, the solutions and the isolated organs were kept at 37°C by thermostatically controlled units throughout the experiment. The samples from venous effluent or from gastric lumen effluent were collected, at the end of every 20 minutes, into chilled test tubes that were immediately stored at –80°C for subsequent measuring experiments.

### Amylase and Lipopolysaccharide Levels

The assays of amylase and lipopolysaccharide (LPS) levels in the serum from AP or control rats were performed based on the manufacturer recommended procedures (Cat. No: C016 for amylase assay kit, Jiancheng Technology, Nanjing, China; and Cat. No: CE32545 for LPS assay kit, Chinese Horseshoe Crab Reagent Co. Ltd., Xiamen, China).

### Assays for Inflammatory Mediators

The levels of interleukin-6 (IL-6) and cytokine-induced neutrophil chemoattractant 1 (CINC1/KC) in the serum of rat and in the venous effluent from the isolated rat stomach were quantified using the rat IL-6 and KC ELISA kits based on the manufacturer recommended protocols (Cat. no: F01731D for rat interleukin 6 ELISA kits; Cat. no: F01723D for rat KC ELISA kits. H-Y Biological Co. Ltd., Shanghai, China). The optical density was determined at 490 nm for absorbance in an enzyme-linked immunoabsorbent assay instrument (Microplate Reader, Model Elx800; BioTek, Winooski, VT, USA). Each specimen was measured three times and the measurement was repeated in 6 rat samples of each group. The data in the venous effluent from the isolated rat stomach was presented with the difference of IL-6 or KC level between the perfusion and the effluent, being considered as the release of IL-6 and KC from the rat stomach.

### Gastrin and Somatostatin Levels in Animal Specimens

Gastrin and somatostatin levels in the animal serum and in the isolated stomach venous effluent were measured using commercially-available gastrin and somatostatin radioimmunoassay Kits (Gastrin Kit: Cat. No. G01PJB, North Institute of Biologic Technology, Beijing, China. Somatostatin Kit: Cat. No. S111013, Second Military Medical University, Shanghai, China). Measurement procedures were based on the manufacturers’ recommendations as described before [21]. As same as above, each specimen was measured three times and the measurement was repeated in 6 rat samples of each group. The data in the venous effluent from the isolated rat stomach was presented with the differences of gastrin or somatostatin level between the perfusion and the effluent, being considered as the release of gastrin and somatostatin from the rat stomach.

### Pepsin and H^+^ Levels in Animal Specimens

The assays of pepsin level in the rat gastric juice and in the gastric lumen effluent from the isolated rat stomach were performed using the manufacturer recommended protocols (Cat. No. A081-1, Jiancheng Technology, Nanjing, China), as [H^+^] in these samples were measured by delta 320 pH-meter (Mettler-Toledo Inc. Zurich, Switzerland) and the readings were then converted to [H^+^].

### Solutions and Chemicals

HU210 [(6aR)-trans-3-(1,1-Dimethylheptyl)-6a,7,10,10a-tetrahydro-1-hydroxy-6,6-dimethyl -6H-dibenzo[b,d]pyran-9-methanol], AM251 [(N-(Piperidin-1-yl)-5-(4-iodophenyl)-1- (2,4-dichlorophenyl)- 4-methyl-1H-pyrazole-3-carboxamide)], were purchased from Tocris (Tocris-Bioscience, Ellisville, MO, USA). Both chemicals were dissolved in a solvent consisted of ethanol, Tween80 and normal saline (NS), volume ratio 1∶1∶18, to concentration 10^−2^M, at 37°C using an ultrasonicator, and then were further diluted in NS to 10^−5^ M, and again to 10^−7^M with perfusion fluid just before use under the conditions that were determined in pilot study [22]. The modified Krebs-Ringer solution was composed of 117.5 mM NaCl, 4.7 mM KCl, 2.4 mM CaCl_2_, 1.1 mM MgCl_2_, 1.1 mM NaH_2_PO_4_, 25 mM NaHCO_3_, 11.1 mM glucose, 0.05% of bovine serum albumin, and 4% dextran. Other agents, if sources were not mentioned above, were purchased from Sigma (Shanghai, China).

### Data Analysis

All data are expressed as mean ± SEM. Student’s t-test or single factor analysis of variance (ANOVA) was performed using the SPSS 13.0 software (SPSS Co. Ltd., Shanghai, China). P-values of <0.05 were considered statistically significant.

## Results

### Results from Experiment *In Vivo*

#### Pathological changes in the pancreas of AP rats

**Figure 1:**
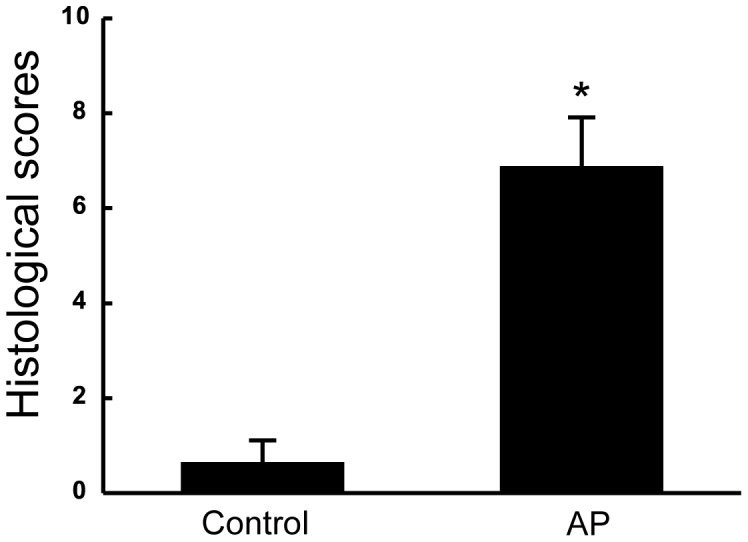
Histological scores for pancreas sections of the control and AP rats. After the induction of acute pancreatitis, rats were sacrificed and organs were harvested. Using the harvested pancreas, histological slides were prepared, stained, examined under microscopy, and scored, as described in MATERIALS AND METHODS. The data are expressed as mean ± SEM (n = 6), *P<0.01 vs control group.

Under light microscopy, it was evident that after treatment with sodium taurocholate, rats developed severe acute pancreatitis with obvious edema, vacuolization and serious necroses in the acinar cells of the pancreatic tissues. And the histological scores in AP rats were much higher than those of the control rats (Fig. 1). Combined with the increased level of amylase activity in the serum of AP rats, the results demonstrated that the AP model replication in rats was successful.

#### Pathological changes in the stomach of AP rats

**Figure 2:**
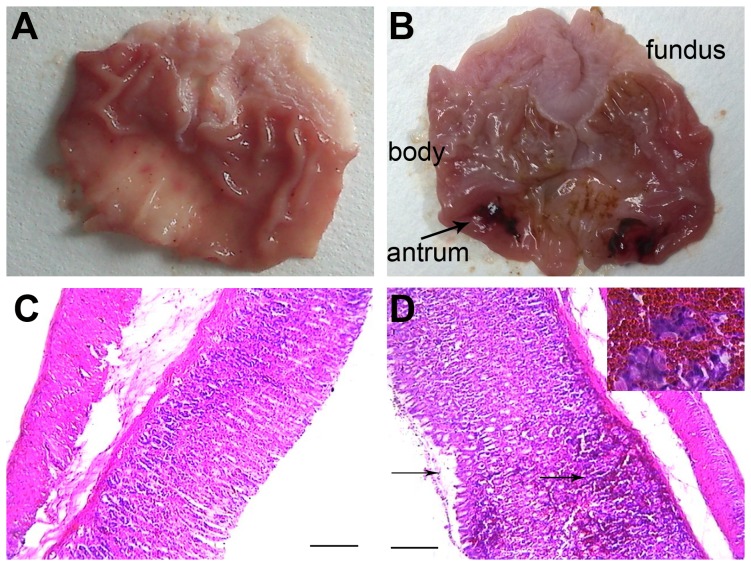
Morphological changes of the isolated stomach from rats with or without experimental acute pancreatitis. (A) Stomach from a control rat. (B) Stomach from an AP rat, showing severe edema and hemorrhages on the gastric antrum (arrowheads). (C) A representative tissue section of the stomach of a control rat, and (D) A representative tissue section of the stomach of an AP rat (hematoxylin and eosin staining, with original magnification ×100; the scale bar = 100 µm). The hemorrhages and mucosal erosions were observed and marked with arrowheads.

In the stomach of the rats with acute pancreatitis, severe pathological changes emerged, exhibiting mucosal edema, erosion and hemorrhages as demonstrated by both macrography (Fig. 2A and 2B) and microscopical examinations (Fig. 2D); and these injuries congregated mainly in the gastric antrum.

#### GeneChip analysis

**Figure 3:**
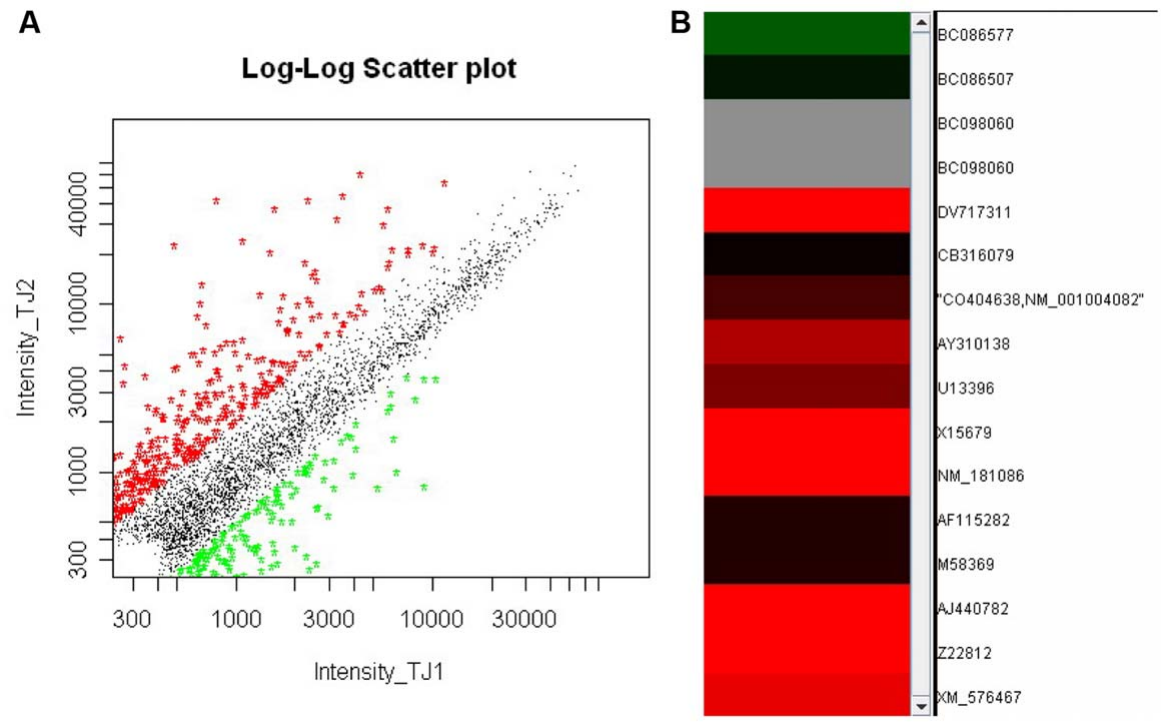
The analyzed expression profile of selected genes in AP rats using a genechip software. (A) The Scatter Plots illustrate the the relative gene expression in pancreas of AP and control rats. Red dots represent genes that were upregulated at least 2-folds (≥2× value of the control, P<0.05), as green dots represent genes downregulated at least 2-folds (≤0.5× value of the control, P<0.05), depicted with the upper and lower boundaries, respectively. (B) The clustering patterns illlustrate the 15 chosen genes (with their Genebank ID) that are closely linked to acute pancreatitis. Red bars symbolize the genes that were upregulated 2-folds or more (P<0.05) and green bars the genes that were downregulated 2-folds or more (P<0.05). Each sample was triplicated and the upregulated genes, alongwith their Gene Name and Genebank ID were singled out and listed in Table 1.

As shown in Fig. 3A, the scatter plots represented genes with two-fold and higher expression were in the upper (red) boundary, while genes with two-fold and lower expression in the lower (green) boundary; and the changed genes closely linked to the acute pancreatitis were shown in the clustering patterns (Fig. 3B). It was obvious that in the expression profile, the genes with significantly differential expressions (≥2-fold, P<0.05) are mainly those which were related with the pancreatic digestive enzymes, inflammatory mediators and the signal transduction pathways, which were singled out and listed with their Gene Name and Genebank ID in Table 1.

**Table 1:** The differentially expressed genes in the pancreas of the rats with acute pancreatitis.

Gene title	Gene symbol	GenbankID	Fold change
Trypsin IV precursor	Pretrypsinogen IV	X15679	4.63
Heat shock 27 kDa protein 1	Hspb1	DV717311	10.58
Interleukin 6 signal transducer	Il6st	AY310138	2.74
Interleukin 1 receptor, type II	Il1r2	Z22812	20.90
Tumor necrosis factor receptor superfamily, member 12a	Tnfrsf12a	NM_181086	15.92
Tyrosine-protein kinase JAK2,(Janus kinase 2)	JAK-2	U13396	2.04
MAP kinase-activated protein kinase 3	Mapkapk3	XM_576467	3.84
Inositol 1,4,5-trisphosphate 3-kinase C	Itpkc	AJ440782	50.24

#### Changes of IL-6, KC and LPS levels in AP serum

**Figure 4:**
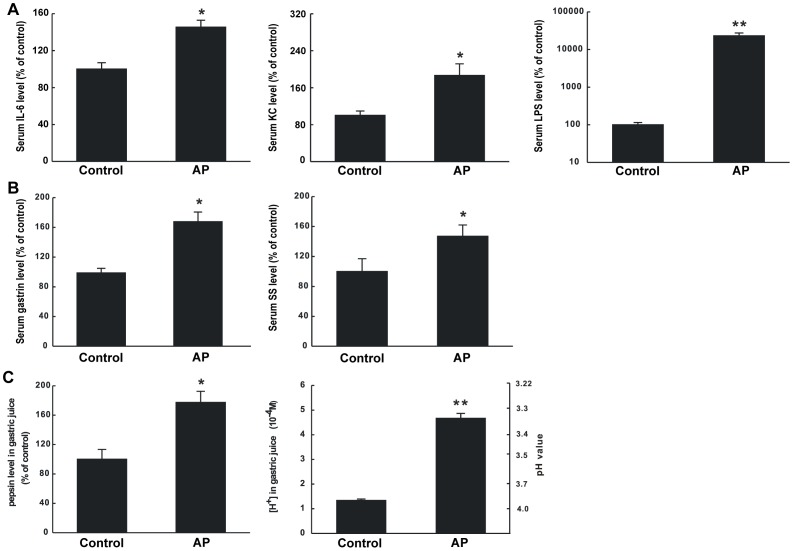
Changes of the components in serum and in gastric juice of rats with experimental acute pancreatitis. (A) IL-6, KC and LPS levels in rat serum. (B) Gastrin and somatostatin levels in rat serum. (C) Pepsin levels and [H^+^] in rat gastric juice. Each specimen was measured three times and data are expressed as mean ± SEM (n = 8). *P<0.05 vs control, **P<0.01 vs control.

Both IL-6 and KC levels in the serum of AP rats displayed significant increases as compared to those of control rats, with upsurges of 145% and 186%, respectively (P<0.05; Fig. 4). A similar but more prominent increase was seen in the LPS level in the serum of AP rats, with an upsurge as much as 231 times of that of the control group (P<0.01; Fig. 4A).

#### Changes of gastrin and somatostatin levels in the serum of AP rats

In the serum of AP rats, gastrin and somatostatin levels increased significantly as compared to those of control rats, with upsurges of 169% and 147%, respectively (in both cases, P<0.05; Fig. 4B).

#### Changes of pepsin levels and [H^+^] in gastric juice of AP rats

To evaluate the changes of gastric exocrine function, assays for pepsin level and [H^+^] were performed by using the gastric juice of AP and control rats. Both pepsin level and [H^+^] in the gastric juice showed a distinct increase in AP rats as compared to those of control rats, with upsurges of 177% and 347%, respectively (Fig. 4C).

#### Expression of CB1 and CB2 receptors in rat pancreas and stomach

**Figure 5:**
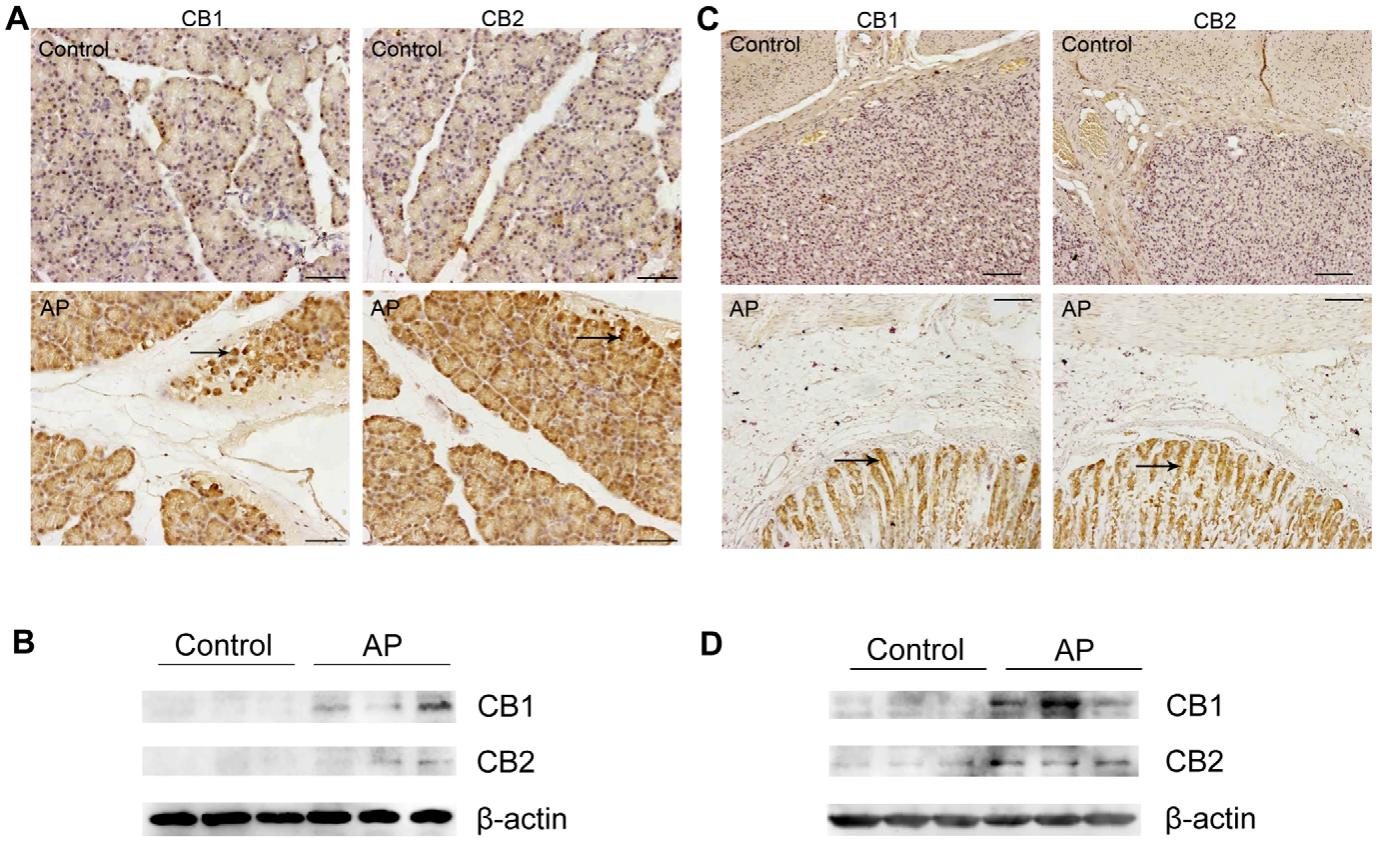
Expression of CB1 and CB2 receptors in rat pancreas and stomach by immunohistochemistry and western blot analyses. (A) Immunohistochemical detection of CB1 and CB2 receptors in rat pancreatic tissue sections, with the arrowheads showing the specific CB1/CB2 staining. (B) Western blot staining of CB1 and CB2 receptors in rat pancreatic tissue lysates. (C) Immunohistochemical detection of CB1 and CB2 receptors in rat stomach tissue sections, with the arrowheads showing the specific CB1/CB2 staining. (D) Western blot staining of CB1 and CB2 receptors in rat stomach tissue lysates. Note that the pancreatic acini and gastric mucosa exhibit increased immunological activity for CB1 and CB2 receptors after the induction of acute pancreatitis. (Original magnification: ×200, and scale bar = 50 µm).

The expression characteristics of CB1 and CB2 receptors in rat pancreas and stomach were investigated. The results demonstrated that the specimens from animals in control group presented only weak immunohistological staining for CB1 and CB2 receptors in the pancreas, whereas specimens from AP rats had exhibited increased expressions of CB1 and CB2 receptors. Mainly, the strong positive signs of brown dyeing clustered in the pancreatic acini (Fig. 5 A arrowheads). The up-regulations of CB1 and CB2 receptors in the pancreatic tissues of AP rats were further demonstrated by western blot analysis and presented in Fig.5 B. The similar expression characteristics of CB1 and CB2 receptors were also found in the stomach of the AP rats, as demonstrated by both immunohistological staining and western blot assay (Fig. 5 C and 5 D). The strong positive signs of brown dyeing were mainly in the gastric mucosa (Fig. 5 C, arrowheads).

### Results from Experiment *In Vitro*

#### Effect of cannabinoids on gastric pathological changes and on gastrin and somatostatin release

**Figure 6:**
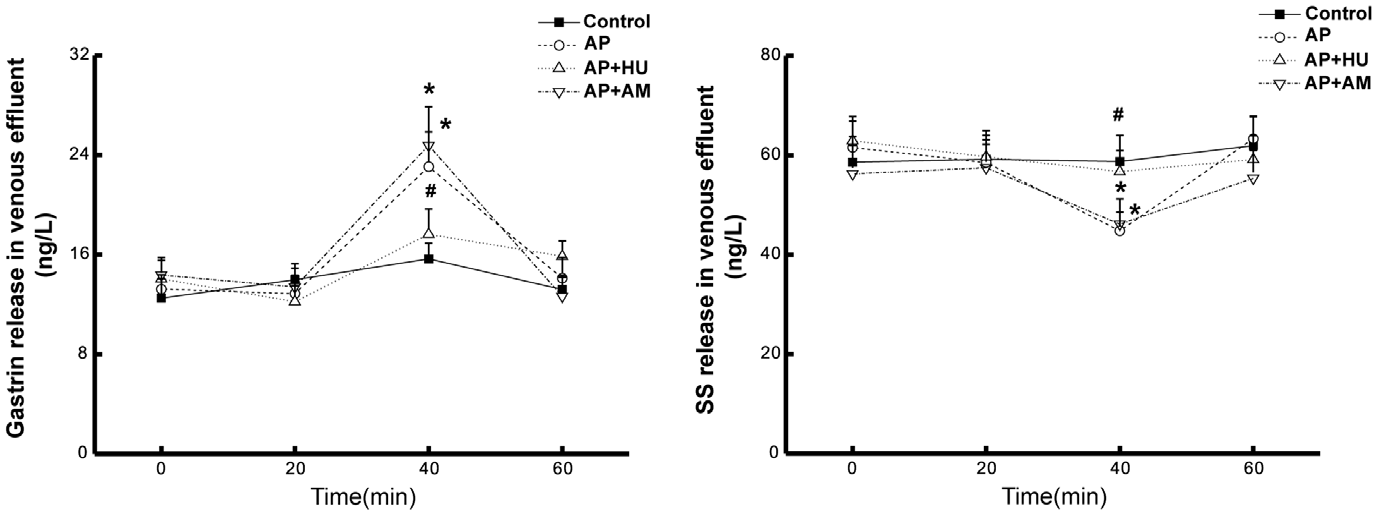
Effects of HU210 and AM251 on gastrin and somatostatin (SS) release from the isolated rat stomach. As described in MATERIALS AND METHODS, the levels of gastrin and somatostatin were measured in the gastric venous effluent of rats during 60 min perfusion with or without the administration of HU210 or AM251. Each specimen was measured three times and data are expressed as mean ± SEM (n = 6). *P<0.05 vs control, #P<0.05 vs those in AP group.

To investigate the effect of CB1 receptor agonist HU210 on the endocrine function of the isolated rat stomach stimulated with AP rat serum, we examined the alterations of gastrin and somatostatin levels in the venous effluent of the stomach, with or without intervention of CB1 receptor agonist HU210 and antagonist AM251. The results showed that compared to the control group, the rat stomach treated with AP serum provoked an increased gastrin release (P<0.05), but a decreased somatostatin release (P<0.05), HU210 reversed the gastrin and somatostatin changes induced by serum of AP rats (P<0.05), while AM251 did not exhibit detectable impact on the release of the two hormones (Fig. 6).

#### Effects of cannabinoids on pepsin activity and [H^+^] in the gastric lumen effluent

**Figure 7:**
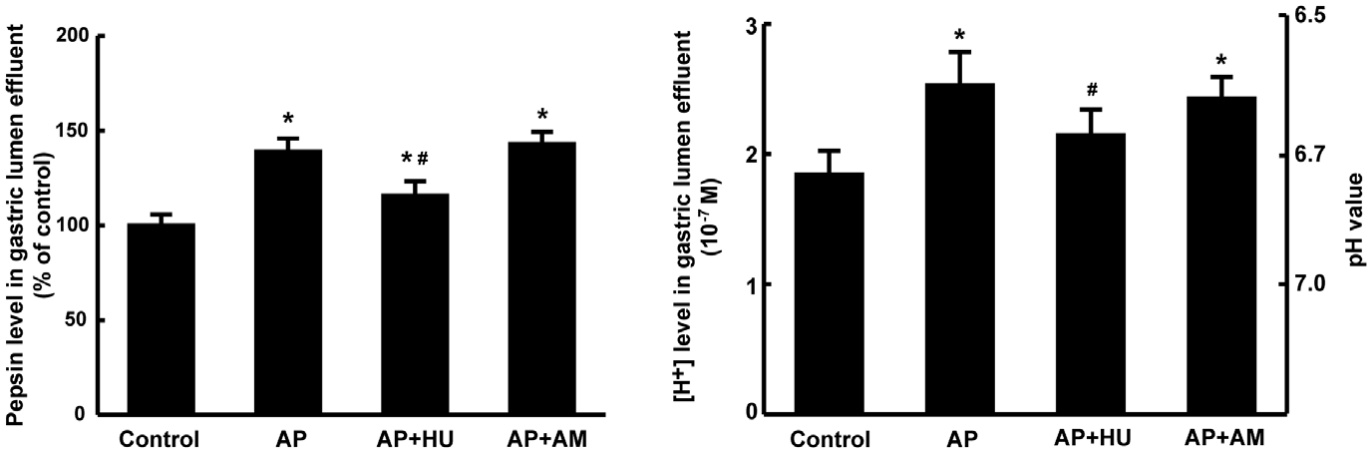
Effects of HU210 and AM251 on pepsin and acid output from the isolated rat stomach. The levels of pepsin and [H^+^] were measured in the rat gastric lumen effluent with or without the administration of HU210 or AM251. Each specimen was measured three times and data are expressed as mean ± SEM (n = 6). *P<0.05 vs control, #P<0.05 vs those in AP group.

The effects of the agents HU210 and AM251 on pepsin activity and [H^+^] in the gastric lumen effluent of the isolated rat stomach were presented in Fig. 7. Compared to the counterparts of the control group, AP serum stimulated the pepsin secretion and acid output in the isolated rat stomach (P<0.05). The intervention of CB1/2 receptor agonist HU210 attenuated the AP serum-induced changes of pepsin secretion and acid output (P<0.05), while the receptor antagonist AM251 failed to exhibit obvious effect on these two parameters.

#### Effects of cannabinoids on the levels of IL-6 and KC in the gastric venous effluent of rats

**Figure 8:**
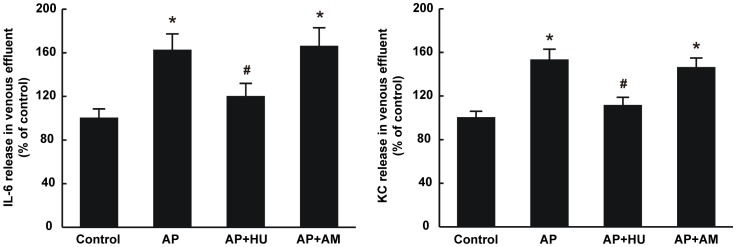
Effects of HU210 and AM251 on the releases of IL-6 and KC from the isolated rat stomach. The levels of IL-6 and KC were measured in the rat gastric venous effluent as described in MATERIALS AND METHODS, Each specimen was measured three times and data are expressed as mean ± SEM (n = 6). *P<0.05 vs control, #P<0.05 vs those in AP group.

After the rats received the treatment of the AP serum, IL-6 and KC levels significantly elevated in the venous effluent from the isolated rat stomach; HU210 reversed the IL-6 and KC changes induced by serum of AP rats (P<0.05), while AM251 had no detectable impact on the levels of the cytokine and chemokine in the venous effluent (Fig. 8).

## Discussion

In clinic, the patients with acute pancreatitis, especially with severe acute pancreatitis, often suffer AGML or stress ulcer, a common complication of AP. The causative factors for stomach injury include, but not limited to, the stress from the inflammatory stimulation which can induce the activation of the locus ceruleus-norepinephrine/sympathetic-adrenal medulla system and the hypothalamus-pituitary-adrenal cortex system. The secretive increases of catecholamines and glucocorticoid hormones are the most important factors of body stress, for these components provoke gastric acid hyper-secretion and blood-flow shifting that cause gastrointestinal mucosal ischemia. Importantly, the ischemia sequentially downgrades the ability of the gastric mucosa to dispose of back-diffusing acid, resulting in a decrease of intramural pH and activation of protease, and subsequent ulceration [3], [4], [23]. Other mechanisms, including oxygen-derived free radicals and some uncertain factors, also play roles in the gastrointestinal injury related with acute pancreatitis.

Previous investigations have found that AP plasma and AP-related ascitic fluid contain a large amount of toxic substances which are harmful to the body [5], [6], [8], causing the damage of the liver, kidney, lung and circulatory system, and gastrointestinal dysfunction, etc. [24]–[28]. Our prior study discovered that the pancreatic acinar cells suffered calcium overload and reduced vitality, as being incubated with AP serum or ascitic fluid [8]. In this study, we first induced experimentally AP in rats and proved the induction of AP animal model was successful by demonstrating the pathological change of pancreatic morphology and the increase of pancreatic enzyme in rat serum after the induction. Upon affirmation of the model, we continued to establish a gene expression profile to illustrate the altered gene expression of pancreatic enzymes and inflammatory mediators, in an attempt to trace the underline genes that played most critical roles in the pathogenesis of AGML associated to AP. And the results from AP and control rats profiled using gene chip analysis were consistent with those of biochemical assays. In addition, we tested if there were beneficial effects of cannabinoid antagonists and/or agonists in the animals with experimental acute pancreatitis.

Based on the aforementioned results, we addressed the question whether gastric secretion, both the endocrine or exocrine functions, would be altered in AP rats. It is known that gastrin stimulates acid output and pepsin secretion, as somatostatin counteracts the effects of gastrin. When gastrin or somatostatin secretion fails to maintain a basic equilibrium, the surplus pepsin and acid release disproportionally, resulting in damages and dysfunctions of the stomach during acute pancreatitis. As demonstrated in this report, we found a significantly raised gastrin level in serum, and elevated pepsin and acid levels in the gastric juice of AP rats, which confirmed that the endocrine and exocrine functions of the stomach were disturbed in the AP model.

Moreover, the circulating activated proteolytic enzymes, vasoactive proteins and endotoxin specific to the pathogenesis of acute pancreatitis may be responsible for AGML as well. Therefore, we explored the effects of the serum from AP rats on the isolated and perfused rat stomach such that the organ could ignore the systemic stress and impacts. The isolated rat stomach stimulated by serum of AP rat not only showed the eye-visible mucosal injury, but also presented a series of biochemical abnormalities, including higher levels of gastrin, cytokine IL-6, chemokine KC, and lower level of somatostatin in the gastric venous effluent, as well as raised pepsin and acid output in the gastric lumen effluent. It is reasonable to infer that there is an imbalance between the aggressive factor and the protective factor of the gastric mucosa during acute pancreatitis. In particular, the increased gastrin, gastric acid output and pepsin jointly play important roles in the pathogenesis of AGML, aggravating the damage of the stomach and triggering vicious cycles during acute pancreatitis.

During the last decade, a number of publications have shown the anti-inflammatory effects of cannabinoids [29]–[32]. Several studies have shown that cannabinoids inhibit gastric acid secretion and reduce the inflammatory cytokines and other mediator in the plasma of animals with AP [33], [34]. Our results not only confirm these earlier discoveries, but also demonstrate that a chemical HU210, presumably a cannabinoid receptor agonist, serve functions in the same way as cannabinoids in reducing the inflammatory cytokines and other mediators, hence ameliorate the symptoms of AP-associated AGML. Interestingly, the results of this study demonstrate that HU210 can attenuate the gastric endocrine and exocrine changes in the isolated rat stomach irritated by AP serum, reverse the abnormally inflated levels of gastrin, gastric acid and pepsin and muffle the effect of these damaging factors. On the other side, HU210 raises the level of somatostatin which inhibits secretion of gastrin and gastric acid, hence exerts protective action on the gastric mucosa.

The outcomes of the study provide harmonic coherence of gene-chip analysis and biochemical assay data using samples from the animal model, suggesting a novel mechanism that the onset of AGML is, at least partly, due to the gastrin, and gastric acid / somatostain imbalance triggered by the toxins in the AP serum; and cannabinoid agonist HU210 restores the equilibrium, hence the protection. The findings support that HU210 is beneficial for treating acute pancreatitis because of its anti-inflammation role and the preventing effect on the AGML related with acute pancreatitis. The results that the CB1 receptor antagonist AM251 fails to play any role in the AP induced gastric damage support our postulation, confirming the positive roles of CB1/2 receptors.

In a prospective experiment to investigate if the proton pump inhibitors (PPIs) can protect animals with experimental acute pancreatitis, we administered omeprazole (OME, i.p., 40 mg/kg weight), a representative PPI agent, to a group of rats at the same time when AP induction was performed. The preliminary results showed that OME increased the survival rate of AP rats (data not shown). However, it may need multicenter study to elucidate if PPIs are beneficial as a therapeutic option in acute pancreatitis of humans.

Taking all above, the results from our experimental investigation reveal that the inflammatory responses and the disturbances of the gastric secretion, both the endocrine and exocrine functions, are the outcomes of acute pancreatitis, and they in turn contribute to the pathogenesis of AGML. Furthermore, the results suggest that cannabinoid HU210, the CB1/2 receptor agonist, has the therapeutic potential for AGML in acute pancreatitis by attenuating inflammation and restoring gastrin/somatostatin equilibrium, and then decreasing the secretion of gastric acid and pepsin. Therefore, our experimental results suggest a novel mechanism in the onset of AGML and new therapeutic values of cannabinoids as supplement of anti-inflammatory therapy in acute pancreatitis.
